# Systematic Review and Meta-Analysis of Tacrolimus versus Ciclosporin as Primary Immunosuppression After Liver Transplant

**DOI:** 10.1371/journal.pone.0160421

**Published:** 2016-11-03

**Authors:** Gorden Muduma, Rhodri Saunders, Isaac Odeyemi, Richard F. Pollock

**Affiliations:** 1 Astellas Pharma Europe Limited, Chertsey, England; 2 Ossian Health Economics and Communications GmbH, Basel, Switzerland; University Hospital Oldenburg, GERMANY

## Abstract

**Background and Aims:**

Several meta-analyses comparing ciclosporin with tacrolimus have been conducted since the 1994 publication of the tacrolimus registration trials, but most captured data from randomized controlled trials (RCTs) predating recent improvements in waiting list prioritization, induction protocols and concomitant medications. The present study comprised a systematic review and meta-analysis of ciclosporin and tacrolimus in liver transplant recipients using studies published since January 2000.

**Methods:**

Searches of PubMed, the Cochrane Library and EMBASE identified RCTs of tacrolimus and ciclosporin as the immunosuppressant in adult primary liver transplant recipients, published between January 2000 and August 6, 2014. A random effects meta-analysis was conducted to evaluate the relative risk of death, graft loss, acute rejection (AR), new-onset diabetes after transplantation (NODAT) and hypertension with tacrolimus relative to ciclosporin at 12 months.

**Results:**

The literature search identified 11 RCTs comparing ciclosporin with tacrolimus. Relative to ciclosporin, tacrolimus was associated with significantly improved outcomes in terms of patient mortality (risk ratio [RR] with ciclosporin of 1.26; 95% confidence interval [95%CI] 1.01–1.58). Tacrolimus was superior to ciclosporin in terms of hypertension (RR with ciclosporin 1.26; 95%CI 1.07–1.47), but inferior in terms of NODAT (RR with ciclosporin 0.60; 95%CI 0.47–0.77). There were no significant differences between ciclosporin and tacrolimus in terms of graft loss or AR.

**Conclusions:**

Meta-analysis of RCTs published since 2000 showed tacrolimus to be superior to ciclosporin in terms of patient mortality and hypertension, while ciclosporin was superior in terms of NODAT. No significant differences were identified in terms of graft loss or AR. These findings provide further evidence supporting the use of tacrolimus as the cornerstone of immunosuppressive therapy in liver transplant recipients.

## Background and Aims

Calcineurin inhibitors (CNIs) ciclosporin and tacrolimus form the cornerstone of immunosuppressive therapy in liver allograft recipients. Ciclosporin was introduced as a novel immunosuppressive agent in 1980, resulting in greatly improved outcomes in patients undergoing liver transplantation [1]. In 1994, two pivotal registration trials of tacrolimus were published in liver transplant recipients showing a significant reduction in the incidence of acute rejection, but no difference in mortality or graft loss compared to ciclosporin at 1 year [2,3]. Ciclosporin was subsequently reformulated into a microemulsion (Neoral, Novartis, Basel, Switzerland) prompting further comparisons of its efficacy relative to both the conventional formulation and tacrolimus [4,5]. More recently, tacrolimus has been reformulated from a twice-daily “immediate-release” formulation (Prograf, Astellas Pharma Inc., Tokyo, Japan) to a once-daily prolonged-release formulation (Advagraf, Astellas Pharma Inc., Tokyo, Japan).

Changes to the formulations of primary immunosuppressive therapies have been accompanied by more widespread use of induction therapies in addition to improvements in waiting list prioritization, donor-recipient matching, and concomitant immunosuppressive medications [6–9]. For instance, the use of induction antibody preparations, while still not universal in liver transplant recipients, has been steadily increasing from 13.3% in 1999 to 26.7% in 2008, a change that has been ascribed to attempts to reduce CNI-induced nephrotoxicity immediately after transplantation [7]. Furthermore, since the tacrolimus registration trials in 1994, the anti-proliferative agent mycophenolic acid (MPA) was approved by the FDA in its prodrug form, mycophenolate mofetil (MMF). As with azathioprine, MMF can reduce nephrotoxicity by sparing the use of CNIs [10]. MMF has been the most prevalent adjunct therapy for more than 10 years. Waiting list prioritization has improved greatly as a result of the introduction and widespread use of the model for end-stage liver disease (MELD) score and the development of risk indices such as the liver donor risk index (LDRI) [8,9]. Finally, mechanistic target of rapamycin (mTOR) inhibitors such as sirolimus and everolimus have been introduced and approved for use in liver transplant recipients, although sirolimus in particular has had a challenging relationship with regulators, with the Food and Drug Administration (FDA) issuing two black box warnings for reported instances of hepatic artery thrombosis in *de novo* patients and increased mortality after conversion from CNIs before the data had undergone close scrutiny [11].

A number of reviews and meta-analyses have been conducted on the relative efficacy of immunosuppressive agents in liver transplantation, including a 1998 review by Busuttil and Holt, a 2006 Cochrane meta-analysis by Haddad *et al*. and a 2014 analysis focusing specifically on patients with hepatitis C virus (HVC) [12–14]. However, many of these analyses include data from older trials of tacrolimus and ciclosporin, predating either the use of ciclosporin microemulsion or the more prevalent use of MMF as the antiproliferative agent in place of the anti-metabolite azathioprine. Given that these and other aspects of routine clinical practice have improved markedly in the years following the tacrolimus registration trials, an assessment of more modern usage of immunosuppressive agents in liver transplant recipients is overdue. Moreover, previous meta-analyses including the original registration trials may have reported an inflated effect size of tacrolimus relative to ciclosporin as a result of the widely-observed “fading of reported effectiveness” phenomenon, in which early studies give yield inflated estimates of effect [15–17]. The present study therefore aimed to identify randomized controlled trials (RCTs) of immediate-release tacrolimus, prolonged-release tacrolimus and ciclosporin published since January 1, 2000 and conduct a meta-analysis of the key outcomes to establish the relative effectiveness of the tacrolimus formulations and ciclosporin in adult patients undergoing liver transplantation.

## Methods

A systematic literature search of the PubMed, Cochrane Library and EMBASE databases was conducted. Search terms were devised using a combination of free-text title and abstract search terms and Medical Subject Heading (MeSH) terms to identify parallel-group randomized controlled trials comparing all formulations of tacrolimus with ciclosporin as the primary immunosuppressive regimen used immediately (i.e. up to one week) after first liver transplantation in adult populations (Table 1). Searches were conducted to identify studies published in peer-reviewed journals between January 1, 2000 and August 6, 2014. Congress proceedings were captured through their inclusion in EMBASE, but were excluded if a full manuscript had not subsequently been published in a peer-reviewed journal. Inclusion was not dependent on the use of specific concomitant immunomodulatory therapies (such as corticosteroids or MMF) or induction protocols (such as basiliximab or thymoglobulin infusions), but studies in which other such therapies or protocols were employed were only included if the practice was used in both trial arms. Azathioprine was exempt from this requirement to align the inclusion criteria with the 2006 meta-analysis from the Cochrane Collaboration, which included a sufficient number of studies to stratify by azathioprine use [13]. No protocol for this analysis was registered before manuscript submission. The analysis and write-up were conducted in line with the Preferred Reporting Items for Systematic Reviews and Meta-Analyses (PRISMA) checklist (S1 File).

**Table 1:** Literature search strategy.

Line #	Search Terms
#1	exp **liver transplantation**/ OR (**liver**:ti,ab,kw AND transplant*:ti,ab,kw)
#2	tacrolimus*:ti,ab,kw OR **Advagraf**:ti,ab,kw OR **Prograf**:ti,ab,kw OR FK506*:ti,ab,kw OR exp **Tacrolimus**/ OR **LCP-Tacro**:ti,ab,kw OR **LCPT**:ti,ab,kw OR **Envarsus**:ti,ab,kw
#3	cyclosporin*:ti,ab,kw OR ciclosporin*:ti,ab,kw OR Neoral*:ti,ab,kw OR Sandimmun*:ti,ab,kw OR exp **Cyclosporin/**
#4	**#2** OR **#3**
#5	**#1** AND **#4**
#6	random*:ti,ab,kw OR placebo*:ti,ab,kw OR blind*:ti,ab,kw
#7	**#6** OR (**clinical**:ti,ab,kw OR **controlled**:ti,ab,kw) AND **trial**:ti,ab,kw
#8	**#7** OR **meta-analysis**:ti,ab,kw
#9	**#5** AND **#8** limited to studies published on or after January 1, 2000

Duplicate studies were removed and screening of the titles and abstracts of the remaining unique studies was performed by two researchers independently (RS and RFP) using the exclusion criteria listed in Table 2. Disagreements between reviewers were then resolved by discussion and full-text versions of the remaining included articles were retrieved from the respective publishers. For each study, data on the following endpoints were extracted where available: patient mortality (the primary outcome), graft loss (excluding death with a functioning graft), acute rejection (including biopsy-confirmed acute rejection and treated acute rejection, where available), chronic rejection, new-onset diabetes after transplantation (NODAT), hypertension, renal dysfunction, hepatocellular carcinoma recurrence, post-transplant lymphoproliferative disorder (PTLD), neurotoxicity and hepatitis C (HCV) recurrence (in patients with existing HCV).

**Table 2:** Study exclusion criteria.

Exclusion criteria
Is not a parallel-group, randomized, controlled clinical trial
Pediatric study
Compares maintenance immunosuppressive medications initiated >1 week after receipt of graft
Does not report endpoints of interest at 12 months
Editorial, case-report, letter, comment or author reply
*In vitro* or animal study

Data extraction from included studies reporting endpoints of interest was conducted independently by two researchers (RS and RFP) into a Microsoft Excel (Microsoft Corporation, Redmond, WA, USA) spreadsheet. For each endpoint, the number of events and number of patients at risk were recorded for each arm, along with the time at which the endpoint was reported and any subgroups reported in the trial. Assessment of potential bias was performed at the study level through assessment of funding sources and disclosures using NA to represent no funding information, low to represent research council or government funding, and high to represent commercial funding. Additionally, the Cochrane Collaboration’s risk of bias tool was used to assess risk of selection bias, performance bias, detection bias, attrition bias and reporting bias in each study [18]. Extracted data were manually entered into Review Manager 5.3.4 (The Nordic Cochrane Centre, The Cochrane Collaboration, Copenhagen, Denmark) and a meta-analysis was performed to calculate average treatment effects across trials using a random effects model as described by DerSimonian and Laird [19]. Specifically, treatment effects were calculated as pooled risk ratios (RR) with 95% confidence intervals using the Mantel-Haenszel method to estimate between-study variation [20]. Where possible, subgroup analyses of patients with HCV were also conducted. Study heterogeneity was then assessed using Chi-square (χ^2^), the *I*^*2*^ statistic described by Higgins and colleagues, and visual inspection of the asymmetry of funnel plots of the RR against the standard error (SE) of the log(RR) [21]. Sensitivity analyses were conducted in studies assessed as having “low” bias potential according to funding sources and author disclosures, and in those studies reporting 3 or more categories scored as “low risk” in the Cochrane Collaboration risk of bias tool. An analysis was also conducted in studies published in 2006 or later on the grounds that 2006 was the year in which EuroTransplant adopted the MELD scoring system for waiting list prioritization (after US adoption in 2002) [22]. The robustness of the results was also tested by examining different outcome measures (odds ratios and risk differences) and different statistical models (Mantel-Haenszel fixed effect model).

## Results

**Fig 1:**
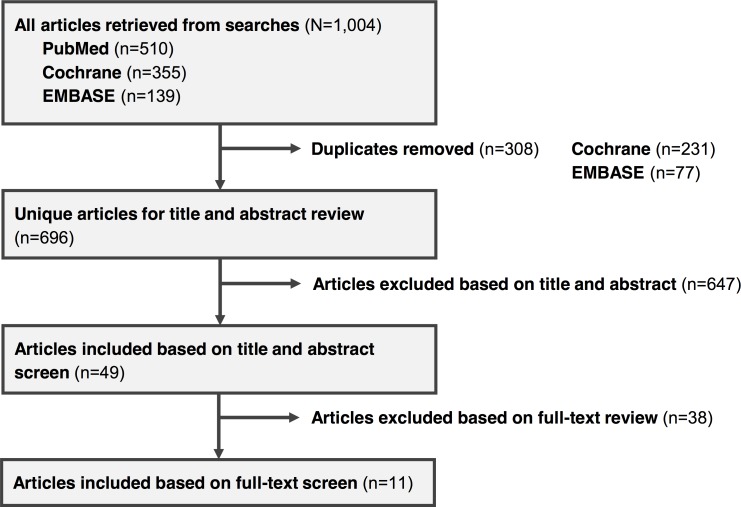
Literature review flow diagram.

The literature searches yielded 1,004 references, of which 308 were duplicates, leaving 696 unique articles for screening. Of the 696 unique articles in the set of results, 102 were included by at least one reviewer based on the article title and abstract. The majority of exclusions during title and abstract screening were due to studies not being randomized, parallel group, controlled trials (reviewers agreed on 272 exclusions on this basis), followed by studies not comparing the treatments of interest (n = 122), not being in first liver transplant recipients (n = 17), or studies excluded for multiple reasons (n = 183, Fig 1).

After resolution of differences between reviewers based on the study title and abstracts, full-text versions of 49 studies were retrieved for further review and data extraction. Of the 49 studies reviewed, 11 were included in the final meta-analysis (Fig 1, Table 3). The 38 studies excluded from the analysis were excluded primarily for not reporting endpoints of interest, for not being published in full in peer-reviewed journals or for not reporting endpoint data 12 months after transplant (S1 Table). With the cut-off date of January 1, 2000, all included studies compared ciclosporin microemulsion (as opposed to the earlier oil-based formulation) with twice-daily, immediate-release tacrolimus. No studies were identified comparing once-daily, prolonged-release tacrolimus directly with ciclosporin; however, one RCT was identified comparing prolonged-release tacrolimus with immediate-release tacrolimus [23]. All RCTs included had some level of industry support, disclosure or involvement. Endpoints with sufficient data for analysis were mortality, graft loss, hypertension, NODAT and acute rejection. All other endpoints for which data were extracted were either reported too infrequently or too inconsistently to include in the meta-analysis.

**Table 3:** Included studies reporting endpoints of interest.

Study	N	Adult study	HCV	Steroids	Anti-proliferative or anti-metabolite	Follow-up (months)	Trial	Funding bias^†^	A	B	C	D	E	F	G
Glanemann 2000	80	Yes	0%	100%	100% MMF	12		NA	+	?	−	−	?	−	+
O’Grady 2002	606	Yes	9.9%	100%	100% AZA	12	TMC	High	+	+	−	+	+	+	+
Greig 2003	143	Yes	32.8%	100%	100% AZA	12		NA	?	?	−	−	+	+	?
Fisher 2004*	99	Yes	37.0%	100%	100% MMF	12		High	?	?	−	−	+	+	?
Martin 2004	79	Yes	100%	100%	100% AZA	12		High	?	+	−	+	?	+	?
Gonzales-Pinto 2005	100	Yes	44%	100%	CicA: 100% AZA	12		NA	−	?	−	−	+	+	?
					Tac: 0% AZA										
Berenguer 2006	90	Yes	100%	100%	CicA: 18% MMF	12		Low	?	?	−	+	?	?	?
					Tac: 15% MMF										
Levy 2006	495	Yes	31.5%	100%	CicA: 43% AZA	12	LIS2T	High	?	?	−	?	+	+	?
					Tac: 41% AZA										
Shenoy 2008	60	Yes	53.3%	100%	CicA: 13% MMF	12		High	?	?	?	?	+	+	?
					Tac: 23% MMF										
Cholongitas 2011	66	Yes	16.7%	CicA: 67%	Not reported	12		Low	+	+	−	?	+	+	?
				Tac: 73.3%											
Levy 2014	351	Yes	100%	CicA: 79.7%	CicA: 12.0% AZA, 53% MMF	12	REFINE	High	+	+	−	+	+	+	?
				Tac: 77.5%	Tac: 11.6% AZA, 41.3% MMF										

AZA, azathioprine; CicA, ciclosporin A; HCV, hepatitis C virus; MMF, mycophenolate mofetil; NA, not available; Tac, tacrolimus. Columns A–G indicate risk of bias assessments using the Cochrane Collaboration risk of bias tool: A, random sequence generation; B, allocation concealment; C, blinding of participants and personnel; D, blinding of outcome assessment; E, incomplete outcome data; F, selective reporting; G, other bias. ‘-‘ indicates high risk, ‘+’ indicates low risk, ‘?’ indicates unclear risk.

* 12-month follow-up data from the Fisher *et al*. study were first published by Haddad *et al*. 2006. These data have been used in the present analysis.^13,28^

^†^ Indication of bias through disclosures or industry funding, NA indicates that no information was provided in the manuscript to assess bias.

In the meta-analysis, study heterogeneity was quantified by the *I*^*2*^ statistic for each outcome. Heterogeneity in terms of the primary outcome of mortality was low, with an *I*^*2*^ value of 0%. Hypertension and acute rejection also exhibited low heterogeneity with *I*^*2*^ values of 0%. NODAT and graft loss endpoints exhibited positive *I*^*2*^ values, of 10% and 53% respectively, representing “low” and “moderate to high” heterogeneity respectively as tentatively defined by Higgins and colleagues [21]. The graft loss analysis yielded a non-significant χ^2^ test result of 8.49 over 4 degrees of freedom (P = 0.08), suggesting that the included studies were relatively homogeneous in terms of their clinical and methodological heterogeneity. In the case of NODAT, all studies were directionally in agreement and the *I*^*2*^ value was driven by a non-significant χ^2^ test outcome of 4.44 over 4 degrees of freedom (P = 0.35).

Bias assessment using the Cochrane Collaboration risk of bias tool revealed a generally poor level of reporting of methodological steps to inform bias assessment, with 30 of 77 assessments having insufficient information to make a definitive judgment on bias potential. The two studies with the lowest assessed risk of bias were amongst the top three largest studies included in the meta-analysis [24,25], with a combined weight of 60.2% in the mortality analysis.

**Fig 2:**
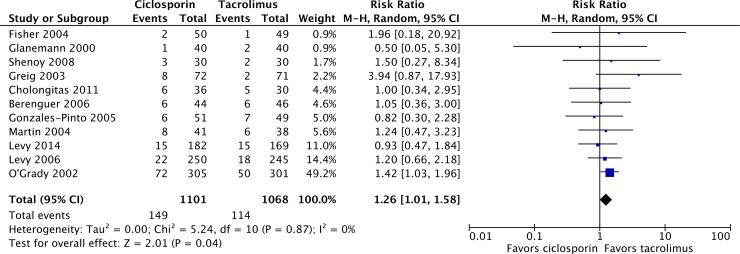
Patient mortality in liver transplant recipients on ciclosporin- or tacrolimus-based immunosuppressive regimens.

**Fig 3:**
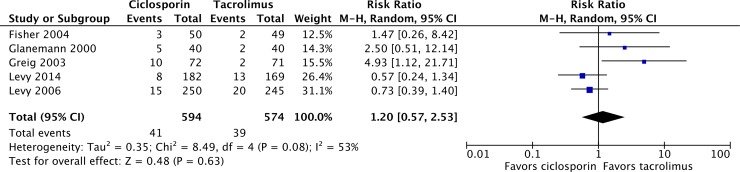
Graft loss in liver transplant recipients on ciclosporin- or tacrolimus-based immunosuppressive regimens.

Over 12 months, 954 of 1,068 patients survived on tacrolimus, compared with 952 of 1,101 on ciclosporin, resulting in a mortality risk ratio (RR) of 1.26 (95% confidence interval [CI] 1.01, 1.58; P = 0.04) with ciclosporin relative to tacrolimus (Fig 2) [24–34]. Graft loss was reported in 5 of the 11 included studies, with a total of 80 events over 12 months of follow-up in 1,168 patients (Fig 3) [25–28,32]. Of the 594 patients on ciclosporin-based immunosuppression, 41 patients experienced graft loss, compared with 39 of 574 patients on tacrolimus, yielding a graft loss RR of 1.20 (95% CI 0.57, 2.53; P = 0.63) with ciclosporin relative to tacrolimus. The most common causes of death in the included studies were sepsis and multiple organ failure, infection, primary non-function of the graft, liver failure or recurrence of liver disease.

**Fig 4:**
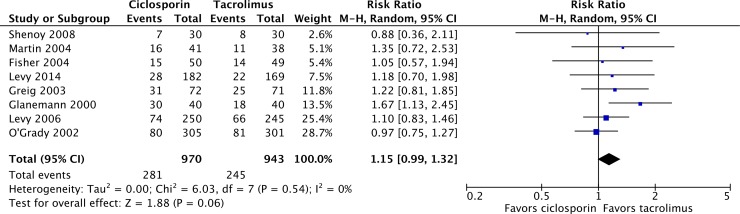
Acute rejection incidence in liver transplant recipients on ciclosporin- or tacrolimus-based immunosuppressive regimens.

**Fig 5:**
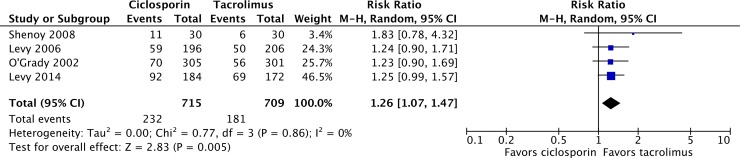
Incidence of hypertension in liver transplant recipients on ciclosporin- or tacrolimus-based immunosuppressive regimens.

Eight of the included studies reported acute rejection at 12 months as an endpoint, with 970 and 943 patients at risk in the ciclosporin and tacrolimus arms respectively [25–29,32,33]. There were a total of 281 acute rejection events in patients on ciclosporin compared with 245 events in patients on tacrolimus, resulting in a risk ratio of 1.15 (95% CI 0.99–1.32; P = 0.06) for ciclosporin relative to tacrolimus (Fig 4). Hypertension at 12 months was reported in four studies, in which 232 of 715 patients on ciclosporin had hypertension after 12 months, compared with 181 of 709 patients on tacrolimus, resulting in a risk ratio of 1.26 significantly in favor of tacrolimus (95% CI 1.07–1.47; P = 0.005; Fig 5) [24,25,32,33]. Shenoy *et al*. defined hypertension as “the need for treatment for the condition” with antihypertensive medication being prescribed at the discretion of the treating physician given a goal blood pressure less than 140/90 mmHg [33]. Levy *et al*. 2006 and O’Grady *et al*. 2002 used definitions of “incidence of treatment for *de novo* hypertension” and “need for antihypertensive therapy” without specifying a threshold and no definition was provided by Levy *et al*. 2014 [24,25,32].

**Fig 6:**
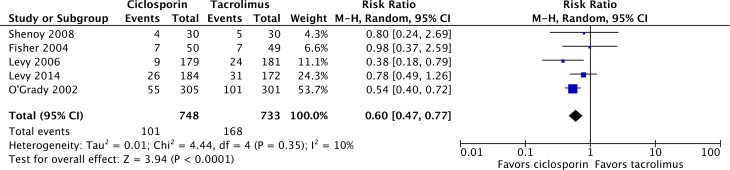
Incidence of new-onset diabetes after transplantation in liver transplant recipients on ciclosporin- or tacrolimus-based immunosuppressive regimens.

Finally, NODAT rate at 12 months after transplantation was reported in five of the studies, with 101 of 748 patients on ciclosporin compared with 168 of 733 on tacrolimus experiencing NODAT after transplantation (Fig 6), resulting in a significant NODAT risk ratio of 0.60 (95% CI 0.47–0.77; P<0.0001) for ciclosporin relative to tacrolimus [24,25,28,32,33]. NODAT definitions included “patients who were not diabetic at baseline [who] were treated for diabetes at 12 months”, “changes from pretransplant diabetic treatment”, and “sustained (>1 month) requirement for oral hypoglycemic agents or an insulin requirement in a patient not already known to be diabetic”. NODAT was reported but not defined in two studies [24,25,28,32,33].

The single study comparing prolonged-release with immediate-release tacrolimus demonstrated non-inferiority with regard to the primary endpoint of biopsy-confirmed acute rejection at 24 weeks (33.7% for immediate-release versus 36.3% for prolonged-release tacrolimus, P = 0.512; treatment difference 2.6%; 95% CI –7.3%, 12.4%), falling within the study’s predefined 15% non-inferiority margin [23]. The study also reported the secondary endpoints of patient and graft survival outcomes at 24 weeks and 12 months. Kaplan–Meier estimated patient survival rates for immediate-release versus prolonged-release tacrolimus were comparable at 12 months at 90.8% versus 89.2% (P = 0.535, treatment difference 1.6%; 95% CI –7.2%, 3.8%), respectively, while graft survival rates were 85.6% versus 85.3% (P = 0.876, treatment difference 0.3%; 95% CI –6.8%, 6.1%). Given that the literature review identified only a single non-inferiority study of prolonged-release tacrolimus, further analysis (such as indirect treatment comparison) was not conducted as part of the present analysis.

### HCV subgroups

Subgroup analyses were conducted in patients with HCV based on the studies unambiguously reporting outcomes specifically in patients whose primary indication for liver transplant was HCV [25,28,29,31,32]. Five studies reported the number of events for patient survival, while four studies reported graft loss in HCV patients. Substantial differences between all patients and the HCV subgroups were observed in terms of the relative incidence of endpoints for which analyses were possible. In the HCV survival analysis, 339 patients of 375 survived on ciclosporin compared with 317 of 355 on tacrolimus, resulting in a weighted RR of 0.91 with ciclosporin relative to tacrolimus, but the finding was not significant (95% CI 0.59–1.41; P = 0.68). Incidence of graft loss in HCV patients was significantly higher in patients treated with tacrolimus than ciclosporin, with 14 graft losses in 291 patients at risk on ciclosporin compared with 27 graft losses in 280 patients at risk on tacrolimus. The risk ratio for graft loss in the HCV subgroup was 0.52 (95% CI 0.28, 0.98; P = 0.04) with ciclosporin relative to tacrolimus.

### Sensitivity analyses

Based on funding status, only two of the studies were classified as being at “low” risk of bias, only allowing analysis of the mortality endpoint in those studies, and showing a reduction in the relative risk of mortality with tacrolimus to a non-significant 1.02 (95% CI 0.36, 3.00) from the significant finding of 1.26 in the main analysis. Selecting only studies reporting a “low” risk of bias in three or more categories in the Cochrane Collaboration risk of bias tool allowed analysis of all included endpoints, albeit based on 1–4 studies (Table 4), down from the 4–11 studies included in the main analyses. Only the hypertension analysis remained significant in the studies with lower risk of bias, with the relative risk increasing to 1.27 (95% CI 1.05, 1.52) from 1.26 in the main analysis. NODAT, which was significantly more common with ciclosporin in the main analysis, failed to reach significance when including only the studies with lower risk of bias, with the 95% CI expanding from 0.47–0.77 in the main anlaysis to 0.38–1.02 in the low bias sensitivity analysis (around pooled risk ratios of 0.60 and 0.62, respectively). The graft loss result failed to reach significance in either the main analysis or the lower bias study analysis, but the pooled risk ratio was directionally opposite from that in the main analysis at 0.67 (compared with 1.20).

**Table 4:** Key sensitivity analyses around the primary analyses.

	Mortality	Graft loss	Acute rejection	Hypertension	NODAT
*Base case*, *RR (95% CI)*	*1*.*26 (1*.*01*, *1*.*58)*	*1*.*20 (0*.*57*, *2*.*53)*	*1*.*15 (0*.*99*, *1*.*32)*	*1*.*26 (1*.*07*, *1*.*47)*	*0*.*60 (0*.*47*, *0*.*77)*
Fixed effects model, RR (95% CI)	1.27 (1.01, 1.59)	1.02 (0.67, 1.56)	1.12 (0.97, 1.29)	1.26 (1.07, 1.48)	0.59 (0.47, 0.73)
Odds ratio, OR (95% CI)	1.31 (1.00, 1.71)	1.23 (0.55, 2.75)	1.19 (0.95, 1.49)	1.40 (1.11, 1.78)	0.53 (0.38, 0.75)
Risk difference, RD (95% CI)	0.02 (0.00, 0.05)	0.02 (−0.03, 0.07)	0.03 (−0.01, 0.08)	0.06 (0.02, 0.11)	−0.08 (−0.13, −0.02)
Low risk of bias according to funding sources and disclosures, RR (95% CI)	1.02 (0.36, 3.00)	—	—	—	—
Three or more categories classified as “low risk” in the Cochrane Collaboration risk of bias tool, RR (n; 95% CI)	1.29 (4; 0.98, 1.96)	0.57 (1; 0.24, 1.34)	1.05 (3; 0.84, 1.27)	1.24 (2; 1.03, 1.50)	0.62 (2; 0.43, 0.88)
Studies published in 2006 or later, RR (n, 95% CI)	1.08 (5; 0.74, 1.57)	0.67 (2; 0.40, 1.12)	1.10 (3; 0.87, 1.39)	1.27 (3; 1.05, 1.52)	0.62 (3; 0.38, 1.02)

CI, confidence interval; OR, odds ratio; NODAT, new-onset diabetes after transplantation; RD, risk difference; RR, risk ratio

A series of sensitivity analyses were conducted in which outcomes were reported in terms of odds ratios and the risk difference and a fixed effects model was used in place of the random effects model used in the main analysis (Table 4). The three significant findings of the main analyses (investigating mortality, hypertension and NODAT) remained significant across sensitivity analyses with the exception of the risk difference and odds ratio analyses used to compare the rates of mortality (P = 0.08 and P = 0.05 respectively). The graft loss analysis was most affected by the switch to a fixed effects analysis, with a fixed effects model yielding a RR of 1.02 (95% CI: 0.67–1.56), reduced from a RR of 1.20 in the random effects analysis. The difference between random- and fixed effects model outcomes is likely to be related to the high heterogeneity observed in the graft loss analysis and may suggest that the random effects estimate, while likely more appropriate than a fixed effects model given the heterogeneity across studies, is not reflective of the true difference between treatments. Changes to the model and the reported outcome measure made no difference to the AR analysis, which remained non-significant across all analyses.

## Discussion

The present meta-analysis showed that, based on randomized controlled trials conducted since January 2000, tacrolimus is significantly more effective than ciclosporin in terms of patient survival and hypertension, with risk ratios of 1.26 (P = 0.04; 95% CI 1.01, 1.58) and 1.26 (P = 0.005; 95% CI 1.07, 1.47), respectively. Conversely, patients on ciclosporin had a lower risk of developing NODAT than those on tacrolimus, with a risk ratio of 0.60 (P<0.0001; 95% CI 0.47, 0.77). No other investigated outcomes significantly differed between ciclosporin and tacrolimus. Analyses of patient survival and graft loss in HCV subgroups were opposite to those in the whole population.

The finding that patient mortality was significantly reduced in patients using tacrolimus relative to ciclosporin is consistent with previous meta-analyses. For instance, in 2006, Haddad and colleagues reported a relative risk of mortality of 0.85 (95% CI 0.73, 0.99) with tacrolimus relative to ciclosporin. Similarly, Haddad *et al*. reported a significantly higher risk of NODAT with tacrolimus relative to ciclosporin with a risk ratio of 1.27, compared to the RR of 0.59 with ciclosporin relative to tacrolimus in the present study. However, the Haddad *et al*. meta-analysis also reported an 18% reduction in the risk of acute rejection with tacrolimus versus ciclosporin, an endpoint around which we identified no significant difference.

Outcomes in the HCV subgroup analysis of graft loss were also in line with many individual RCTs and a 2014 meta-analysis by Liu and colleagues [14]. In terms of individual studies, Villamil *et al*. reported findings from a subgroup of the LIS2T trial, in which other outcomes such as recurrent fibrosis and the proportion of patients with elevated alanine aminotransferase (ALT) levels were also improved in HCV patients treated with ciclosporin relative to tacrolimus [35]. The mechanism for this is relatively well understood; while both tacrolimus and ciclosporin are CNI inhibitors, the mechanism of inhibition is distinct, with ciclosporin blocking efficient HCV replication *in vitro* by binding to regulators of the HCV RNA polymerase, independently of its immunosuppressive effect. Tacrolimus, conversely, binds to FK506 binding proteins, which are not required for HCV replication [36]. The recent meta-analysis by Liu and colleagues reported a graft loss risk ratio of 1.05 (95% CI: 0.83–1.33) with tacrolimus relative to ciclosporin, which matches the present study directionally albeit with a much smaller, non-significant preference toward ciclosporin. However, the short time periods over which RCTs are typically conducted (relative to the natural course of post-transplant HCV) may not be sufficient to capture longer-term differences in patient and graft survival between tacrolimus and ciclosporin that have been observed in registry analyses and extension studies. For instance, a 2011 retrospective analysis of 8,809 liver transplant recipients in the United Network for Organ Sharing (UNOS) database showed three-year unadjusted patient survival rates of 79.9% and 76.8% with tacrolimus and ciclosporin, respectively, and graft survival rates of 75.0% and 71.5% [37]. Significantly higher incidence of mortality was also reported with ciclosporin relative to tacrolimus in a 5 year follow-up to the tacrolimus registration trials, with 21.1% mortality at 5 years in tacrolimus-treated patients compared with 39.5% in the ciclosporin group (p = 0.041) [38]. The 12-month data captured in the present meta-analysis may therefore not be adequate to estimate the longer-term efficacy of the respective immunosuppressive regimens in HCV patients.

Incidence of NODAT in HCV patients was not reported in a sufficient number of studies to conduct a meta-analysis of the relative risks with tacrolimus and ciclosporin. Nevertheless, data from the included LIS2T RCT showed that risk of NODAT was significantly higher in HCV patients treated with tacrolimus relative to those on ciclosporin [32]. Specifically, NODAT was reported as an adverse event in 18 patients (7%) in the ciclosporin group relative to 35 patients (14%) in the tacrolimus group (p<0.02) [32]. The higher incidence of NODAT with tacrolimus relative to ciclosporin in HCV patients has been corroborated by observational data published in 2007 [39]. Careful consideration should therefore be given to the choice of immunosuppressive regimen in HCV patients [39].

While hypertension was not investigated in the Cochrane meta-analysis, the present study showed that tacrolimus would be expected to result in reduced incidence of hypertension relative to ciclosporin, a finding that is in line with the results of a meta-analysis comparing tacrolimus with ciclosporin in recipients of other solid organ grafts [40]. The RCTs identified in the systematic review either did not provide sufficiently homogeneous definitions or did not report sufficient data to conduct analyses of renal dysfunction, hepatocellular carcinoma recurrence, PTLD, or neurotoxicity. Four of the included studies reported “renal events” [24,25,30,34] and showed a general trend towards reduced incidence of events with tacrolimus relative to ciclosporin.

While the quality of reporting in the included trials made accurate bias assessments difficult, the two studies with the least apparent potential for methodological bias coincided with studies that carried significant weighting in the primary analysis of mortality, namely the studies published by O’Grady *et al*. and Levy *et al*. 2014, which were associated with weightings of 49.2% and 11.0%, respectively [24,25]. One other potential source of bias in the present meta-analysis is that of publication bias, namely omitting studies that have not been published or have only been published in abstract form. The former is extremely difficult to address and in the latter case, the present study protocol did not include abstract-only publications. The rationale for ttheir exclusion was that all included studies required critically appraisal and methodological assessment based on a detailed exposition of study methodology, which is almost universally lacking in abstracts and congress proceedings. Furthermore, abstracts that go on to be published in full often present different outcomes from the results presented in the final publication, possibly as a result of methodological changes made in response to peer review or due to incorporation of additional data [41–43]. Nevertheless, the results of the meta-analysis should therefore be interpreted with this caveat in mind.

The literature search identified three studies that met all inclusion criteria with the exception of the time after transplant at which results were reported [44–46]. All three studies reported endpoint data at 3 months after transplant. Two of the three studies reported hypertension as an endpoint, one of which reported a significant reduction with tacrolimus relative to ciclosporin, in agreement with the findings of the meta-analysis [46]. The other study reported no significant difference in hypertension and no individual significant differences were reported in any of the other endpoints investigated in the meta-analysis, possibly as a result of the shorter follow-up time.

All trials included in the meta-analysis compared ciclosporin microemulsion with twice-daily, immediate release tacrolimus. One randomized controlled trial of once-daily tacrolimus was identified in the literature. The study, published by Trunečka *et al*. in 2010, compared twice-daily tacrolimus with once-daily tacrolimus in 475 liver transplant recipients over 24 weeks, with a 12-month extension period. The study was excluded from the meta-analysis as there was no comparison with ciclosporin and an indirect treatment comparison was not performed on the grounds that only this single non-inferiority study was identified. However, given the effectiveness of tacrolimus relative to ciclosporin in a general liver allograft recipient population, consideration should be given to the exact tacrolimus formulation to be used in liver transplant recipients. The primary endpoint of the Trunečka study was powered to demonstrate non-inferiority (within a 15% margin) of once-daily tacrolimus relative to twice-daily in terms of biopsy-confirmed acute rejection (BCAR). The study reported that once-daily tacrolimus reached non-inferiority in terms of BCAR at 24 weeks and showed comparable outcomes in terms of graft and patient survival at 24 weeks and after 12 months of follow-up.

While the Trunečka study represents the only randomized controlled trial of once-daily versus twice-daily tacrolimus, recent analyses of data from the European Liver Transplant Registry (ELTR) show that, in routine clinical practice in Europe, prolonged-release tacrolimus may be superior to immediate-release tacrolimus in terms of graft survival outcomes [47]. Kaplan-Meier analyses of 810 propensity-score matched patients (270 patients on prolonged-release tacrolimus, 540 on immediate-release tacrolimus) showed patient survival rate to be 89% with once-daily tacrolimus after three years compared with 82% with twice-daily tacrolimus (p = 0.003) and graft survival rate to be 89% with once-daily compared with 81% with twice-daily (p = 0.002) [41]. This outcome may have been driven by the demonstrable improvements in patient adherence to immunosuppressive therapy associated with reduced pill burden in patients using a once-daily tacrolimus regimen [48–50]. Further research would be required to establish the veracity of these data as the ELTR was not designed specifically to evaluate immunosuppressive regimens.

Drivers of effectiveness in routine clinical practice, such as adherence, would be much less likely to manifest in a controlled trial environment [51], but should nevertheless be considered when evaluating the relative effectiveness and cost-effectiveness of therapies in which the responsibility for timely and accurate dosing lies with the patient. While RCTs, by design, provide treatment effect estimates that are less subject to bias from confounding variables than non-randomized studies, there are a number of factors that can limit the generalizability of RCT findings. Notably, stringent inclusion/exclusion criteria and relatively small enrollment can restrict the applicability of the findings to other patient groups, while relatively short follow-up periods limit the ability to understand the relative longer-term implications of the study treatments. These factors reinforce the notion that, especially in situations where RCT data are sparse or the available RCTs are not sufficiently long to capture meaningful differences between treatments, decision-making should be informed by all available evidence, including that derived from suitably robust analyses of non-randomized data such as that recorded in the ELTR [52]. Recent studies have reported quantitative approaches to combining data from randomized and non-randomized studies using Bayesian hierarchical models and adjusting study estimates for potential confounders using differences in patient characteristics between study arms [53]. This could provide an avenue for further research on the relative effectiveness of ciclosporin and tacrolimus formulations in liver transplant recipients.

The present analysis showed that, despite numerous changes in other aspects of routine care for liver transplant recipients since the tacrolimus registration trials in 1994, tacrolimus remains superior to ciclosporin in liver transplant recipients with respect to mortality and hypertension. Emerging data from registry studies show that prolonged-release tacrolimus results in improved patient outcomes relative to immediate-release tacrolimus, a finding that may be driven by relative improvements in adherence and reduced intra-patient variability [42,44].

## Conclusions

Meta-analysis of randomized controlled trials published since 2000 showed that tacrolimus is superior to ciclosporin in terms of mortality and hypertension in liver transplant recipients while ciclosporin resulted in significantly lower incidence of NODAT than tacrolimus. No significant differences were identified in terms of acute rejection or graft loss. Further research would be required to establish the efficacy of prolonged-release tacrolimus relative to immediate-release tacrolimus and ciclosporin in liver transplant recipients, given the emergence of new data from routine clinical practice.

## Supporting Information

S1 File PRISMA checklist. (DOC)

S1 Table Reasons for study inclusions and exclusions after title and abstract screening. (DOCX)
