# Supplementary material for: Systematic Review and Meta-Analysis of Tacrolimus versus Ciclosporin as Primary Immunosuppression After Liver Transplant
Source: PLoS One. 2016 Nov 3;11(11):e0160421. doi: 10.1371/journal.pone.0160421 (PMC5094765; doi:10.1371/journal.pone.0160421)
Supplement: S1 Table — (DOCX) [file pone.0160421.s002.docx]

S1 Table Reasons for study inclusions and exclusions after title and abstract screening

| **Study** | **Inclusion or reason for exclusion** |
| --- | --- |
| Glanemann M *et al.* Transplant Proc. 2000;32(3):522-3. | Included |
| O'Grady JG *et al.* Lancet. 2002;360(9340):1119-25. | Included |
| Greig P *et al.* Liver Transpl. 2003;9(6):587-95. | Included |
| Fisher RA *et al.* Clin Transplant. 2004;18(4):463-72. | Included |
| Martin P *et al.* Liver Transpl. 2004;10(10):1258-62. | Included |
| González-Pinto IM *et al.* Transplant Proc. 2005;37(4):1713-5. | Included |
| Berenguer M *et al.* Liver Transpl. 2006;12(5):762-7. | Included |
| Levy G *et al.* Liver Transpl. 2006;12(10):1464-72. | Included |
| Shenoy S *et al.* Liver Transpl. 2008;14(2):173-80. | Included |
| Cholongitas E *et al.* Clin Transplant. 2011;25(4):614-24. | Included |
| Levy G *et al.* Am J Transplant. 2014;14(3):635-46. | Included |
| Alvarez F *et al.* Transplantation. 2000;69(1):87-92. | No tacrolimus arm |
| Loinaz C *et al.* Transplant Proc. 2001;33(7-8):3439-41. | Does not report endpoints of interest at 12 months |
| Mühlbacher F *et al.* Transplant Proc. 2001;33(1-2):1339-40. | Does not report endpoints of interest at 12 months |
| Burroughs A *et al.* J Hepatol. 2002;36(Suppl 1):26. | Data from other included study (TMC trial; O'Grady *et al.*) |
| Chen JW *et al.* Transplant Proc. 2002;34(5):1507-10. | Does not report endpoints of interest at 12 months |
| Therapondos G *et al.* Liver Transpl. 2002;8(8):690-700. | Does not report endpoints of interest at 12 months |
| Timmermann W *et al.* Transplant Proc. 2002;34(5):1516-8. | Does not report endpoints of interest at 12 months |
| Trull A *et al.* Liver Transpl. 2002;8(3):224-32. | Does not report endpoints of interest at 12 months |
| Jara P *et al.* J Pediatr Gastroenterol Nutr. 2002 34(4): 436. | Pediatric study |
| Rodeck B *et al.* J Hepatol. 2002 36(Suppl 1): 31. | Pediatric study |
| Grazi GL *et al.* Am J Transplant. 2004;4(Suppl 8):268. | Data from other included study (LIS2T trial; Levy *et al.* 2006) |
| Levy G *et al.* Transplantation. 2004;77(11):1632-8. | Data from other included study (LIS2T trial; Levy *et al.* 2006) |
| Hardinger KL *et al.* Transplantation. 2004;78(2):377-8. | No full text available |
| Lerut J *et al.* Transplantation. 2004;78(2):173. | No full text available |
| Moench C *et al.* 3rd International Congress on Immunosuppression 2004 San Diego, CA USA. | No full text available |
| Bansal S *et al.* Hepatology. 2004;40(4 Suppl 1):476A. | Pediatric study |
| Kelly D *et al.* Lancet. 2004;364(9439):1054-61. | Pediatric study |
| O'Grady JG Hepatology. 2004;40(4 Suppl 1):551A. | Publication of other included study (TMC trial; O'Grady *et al.*) |
| Tanaka K *et al.* Liver Transpl. 2005;11(11):1395-402. | Data from other included study (LIS2T trial; Levy *et al.* 2006) |
| Jonas S *et al.* Int Immunopharmacol. 2005;5(1):125-8. | Does not report endpoints of interest at 12 months |
| Villamil F *et al.* Transplant Proc. 2006;38(9):2964-7. | Data from other included study (LIS2T trial; Levy *et al.* 2006) |
| Baiocchi L *et al.* Transpl Int. 2006;19(5):389-95. | Does not report endpoints of interest at 12 months |
| Northup PG *et al.* Hepatology. 2006;44(4 Suppl 1):412A-413A. | Does not report endpoints of interest at 12 months |
| Firpi RJ *et al.* Hepatology. 2006;44(4 Suppl 1):421A. | No full text available |
| Jain A. Liver Transpl. 2006;12(10):1452-4. | No full text available |
| Levy G *et al.* Liver Transpl. 2006;12(11):1640-8. | No tacrolimus arm |
| O'Grady JG *et al.* Am J Transplant. 2007;7(1):137-41. | Does not report endpoints of interest at 12 months |
| Lilly L *et al.* Hepatology. 2008;48(4 Suppl):548A-549A. | Data from other included study (REFINE trial; Levy *et al.* 2014) |
| Ishigami M *et al.* Liver Transplant. 2008;14(7 Suppl 1):S179. | Does not report endpoints of interest at 12 months |
| Lerut J *et al.* Ann Surg. 2008;248(6):956-67. | No ciclosporin arm |
| Montalti R *et al.* Liver Transplant. 2008;14(7 Suppl 1):S226. | No tacrolimus arm |
| Lilly L *et al.* Hepatology. 2009;50(4 Suppl):1021A. | Data from other included study (REFINE trial; Levy *et al.* 2014) |
| Trunečka P *et al.* Am J Transplant. 2010;10(10):2313-23. | No ciclosporin arm |
| Aguilera V *et al.* J Hepatol. 2010;52(Suppl 1):S43. | No full text available |
| Marín Gómez LM *et al.* Hepatogastroenterology. 2011;58(106):532-5. | Does not report endpoints of interest at 12 months |
| Levy G. Liver Transplantation. 19th Annual International Congress of the ILTS 2013 Sydney, NSW Australia. | Data from other included study (REFINE trial; Levy *et al.* 2014) |
| Cillo U *et al.* Liver Transplantation. 19th Annual International Congress of the ILTS 2013 Sydney, NSW Australia. | No ciclosporin arm |
| Klintmalm GB *et al.* Am J Transplant. 2014;14(8):1817-27. | No ciclosporin arm |
